# Unilateral Concomitant Antrochoanal Polyp and Fungus Ball in Maxillary Sinus: A Case Report

**DOI:** 10.7759/cureus.19844

**Published:** 2021-11-23

**Authors:** Ibrahim Issa, Derar Al-Domaidat, Adel Danish, Ro'a Al-shaikh Hasan, Hadir Elseidi

**Affiliations:** 1 Otolaryngology - Head and Neck Surgery, Fakeek Care, Jeddah, SAU; 2 Otolaryngology - Head and Neck Surgery, Fakeeh Care, Jeddah, SAU; 3 Pathology, Fakeeh Care, Jeddah, SAU; 4 Internal Medicine, Private Clinic, Jeddah, SAU

**Keywords:** chronic rhinosinusitis, endoscopic sinus surgery (fess), maxillary mycetoma, fungus ball, antrochoanal polyp

## Abstract

Antrochoanal polyp (ACP) are benign lesions that arise from the maxillary sinus, grow into the maxillary sinus, and reach the choana, nasal obstruction being their main symptom. A fungal ball (FB) is a dense accumulation of extra mucosal fungal hyphae, usually within one sinus, most commonly the maxillary sinus. We describe a case of a 38-year-old male with a concomitant unilateral maxillary FB and ACP which was surgically excised by endoscopic sinus surgery.

## Introduction

Antrochoanal polyps are benign lesions that arise from the mucosa of the maxillary sinus, grow into the maxillary sinus, and reach the choana, nasal obstruction being the main symptom [1]. A fungal ball (FB) is a dense accumulation of extra mucosal fungal hyphae, usually within one sinus, most commonly the maxillary sinus [2]. Although the most common organism in fungal balls is Aspergillus, the cultures are often negative and other fungal species have also been identified [2]. Fungal balls are seen more commonly in immunocompetent, middle-aged, and elderly females, often with a history of previous dental procedures, especially dental fillings [2].

## Case presentation

A 38-year-old male with a three-year history of slow, progressive right-sided nasal obstruction came to the ear nose and throat (ENT) clinic. He had a history of dental filling procedures for one right upper tooth cavity a year and a half back. It was associated with right-sided facial fullness and pain as well as occasional purulent nasal discharge mainly from the right side with post-nasal secretions. He had no change in smell. His past medical history was free of any co-morbidities, his past surgical history was negative for surgeries or traumas. Before his complaint that started three years ago, he had only complained of minimal intermittent alternative nasal obstruction which did not affect his daily life or sleep.

On physical examination, he had a nasal polyp descending lateral to the middle turbinate and running posteriorly. In addition, he had nasal septal deviation to the right side. On the left side, no polyps or discharge were seen. Throat examination showed clear post nasal drip and no polyps could be seen. His head and neck exams were unremarkable. Endoscopic nasal examination showed a protruding soft glistening polyp with no obvious dilated vessels in the right middle meatus extending toward the posterior choana with severe septal deviation to the right side, endoscopy on the left side showed the choanal part of the right antrochoanal polyp (ACP) and absence of any polyps in the left nasal cavity.

**Figure 1:**
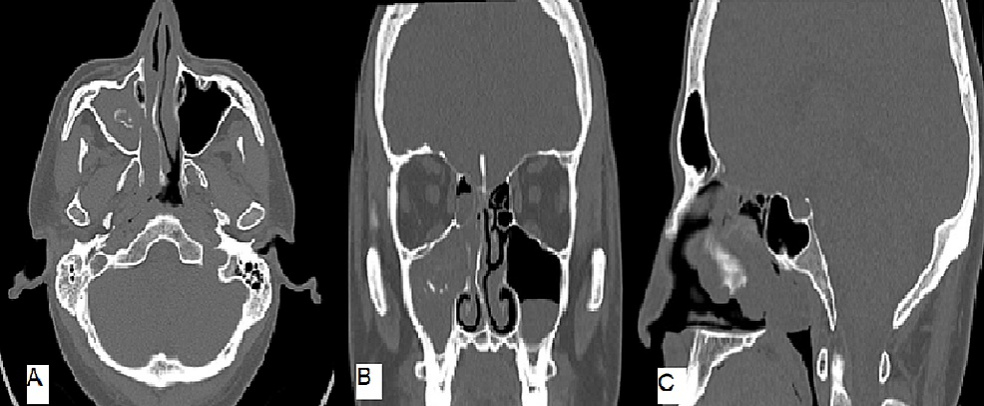
Computed tomography images of paranasal sinuses, showing soft tissue opacification within the right maxillary sinus with extension into the nasal cavity and choana along with the central area of calcific density seen within the right maxillary sinus, A: Sagital view, B: Coronal view, C: Axial view.

Computed tomography (CT) of the nose and paranasal sinuses revealed a large polypoidal soft tissue mass obliterating the right nasal cavity and extending posteriorly to the right choana. Besides, the right maxillary sinus showed complete soft tissue obliteration with a central area of calcific density seen within the right maxillary sinus (Figure 1).

**Figure 2:**
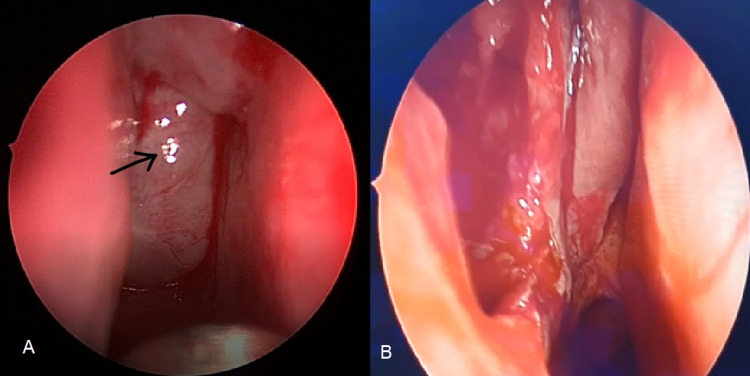
Intraoperative endoscopic pictures, A: Right-sided soft glistening polyp extending posteriorly and completely obstructing the right choana (black arrow). B: At the end of the procedure and performing complete polypectomy and wide middle meatus antrostomy.

The patient was sent for surgery under general anesthesia. At first, septoplasty was done, followed by right-sided Functional Endoscopic Sinus Surgery (FESS) (Figure 2). An uncinectomy with a right wide middle meatal antrostomy was then performed, then polypectomy was done. After that, the maxillary sinus was noticed to be full of cheesy and clay-like debris and a fungal ball was noted in the anteroinferior wall of the maxillary sinus. The pedicle of the polyp was attached to the posterolateral wall of the right maxillary sinus. The origin site of the polyp was removed using giraffe forceps and curved debriders. Irrigation and wash of the maxillary sinus was done to clear the debris. Opening of bulla ethmoidalis on the right side was also done but did not show any disease within it.

**Figure 3:**
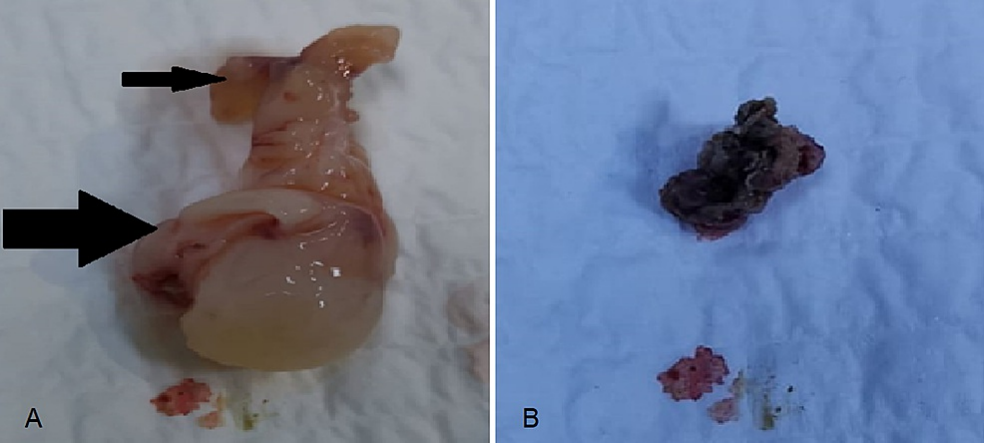
A: The antrochoanal polyp after excision showing its 2 components: the antral part which is almost always cystic and single (thin arrow) and the solid nasal component (thick arrow). B: Fungal ball after removal from maxillary sinus which showed characteristics of rubbery cheesy, clay-like material.

**Figure 4:**
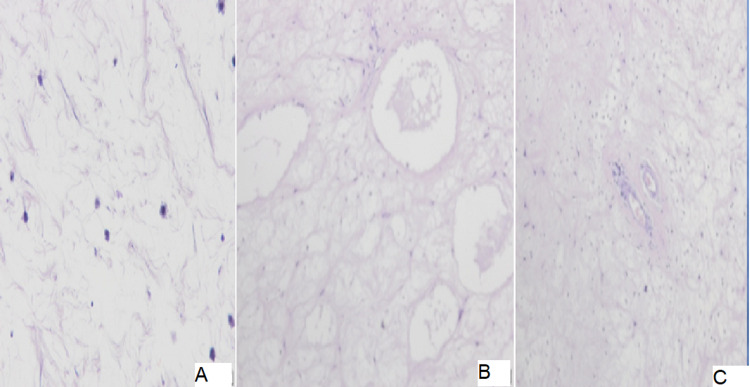
The histology of the first specimen (the right-sided nasal polyp) showed benign ciliated pseudo-stratified columnar epithelium composed of proliferated mucus salivary glands in markedly oedematous vascular chronically inflamed stroma and rich in eosinophils with no fungi or granuloma or malignancy. A, B, and C showing different cuts.

**Figure 5:**
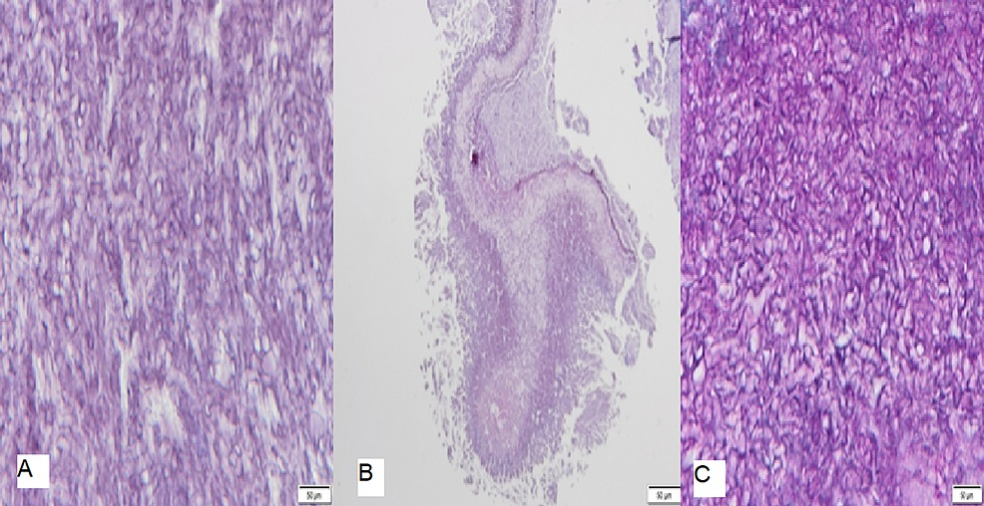
A and B: H/E routine stain shows Ball of overlapping closely packed numerous fungal hyphae with the absence of any eosinophils within it. C: PAS-positive basement membrane of fungal hyphae which are septated and true branched with acute angles consistent with aspergillus

The excised samples (Figure 3) were sent in two different specimens for histopathological examination. The first of them, the right nasal polyp, was consistent with histology of antrachoanal polyp (Figure 4) a benign ciliated pseudo-stratified columnar epithelium and composed of proliferated mucus salivary glands in markedly oedematous vascular chronically inflamed stroma and rich in eosinophils with no fungi or granuloma or malignancy. The second specimen, the right maxillary sinus content, was consistent with aspergillus (Figure 5); there were many colonies and masses of numerous closely packed fungal septate hyphae with positive periodic acid shift (PAS) test and negative vascular invasion. 

The postoperative course was uneventful. Complete resolution of the main patient’s symptoms was achieved after surgery. No recurrence was noted in eight weeks follow-up visits post-operatively.

Tissue culture was not sent because it was not expected to find fungal elements during surgery. However, it should be stressed that even if most fungal balls are made of Aspergillus, cultures are often negative [2].

## Discussion

Fungal ball of the paranasal sinuses is defined as the non-invasive accumulation of dense fungal concernments in sinonasal cavities, most often the maxillary sinus. Affected individuals tend to be older and immunocompetent. Clinical presentation is non-specific and the diagnosis is usually suspected on imaging studies. Surgical treatment, usually through an endonasal endoscopic approach, is curative [2].

The diagnosis of the fungal ball is based on the following features: radiological findings of sinus opacification often with areas of hyperattenuation, cheesy or clay-like debris within the sinus, accumulation of fungal hyphae without evidence of tissue fungal invasion seen microscopically, non-specific chronic inflammation of the sinus and the absence of eosinophil predominance, granuloma or allergic mucin [3].

In 2004, Kaplan et al. investigated the etiology of complete unilateral maxillary sinus opacification in 64 patients [4]. All patients in the series were diagnosed with one of the following: chronic rhinosinusitis (CRS) (without polyps; 42%), mucocele (25%), CRS with nasal polyps (18%), inverted papilloma (10%), or mycetoma (3%).

ACPs are benign lesions that arise from the mucosa of the maxillary sinus, grow into the maxillary sinus, and reach the choana, and nasal obstruction is their main symptom. It comprises 4% to 6% of all nasal polyps and is usually unilateral [5]. One-sided nasal obstruction and discharge are the most common presentation [6]. Computer tomography (CT) scan and nasal endoscopy are the appropriate diagnostic tool [7].

In terms of management, this does not change for both FB and ACP. Several operative procedures have been described in the literature for a fungal ball, for instance, the traditional method introduced by Caldwell- Luc and the more modern techniques of functional endoscopic surgery.

Nowadays, endoscopic sinus surgery is the preferred technique for FB as well as ACP, because postoperative recurrence is lower than other surgical techniques.

The clinical presentation of both, ACP and FB, are very similar. They both occur in immunocompetent people. In addition, they are mostly unilateral diseases and usually present with unilateral nasal obstruction and discharge as well as unilateral facial fullness and pressure. On radiology, they both come with fully opacified maxillary sinus on one side. In terms of management, ACP and FB are both best treated nowadays with complete surgical excision aided by endoscopic sinus surgery. Despite all of these similarities, ACP and FB do not share the same pathophysiology of origin and their simultaneous occurrence is extremely rare in literature. This article describes a third case ever reported in English literature of this rare entity. It shows the difficulty of pre-operative diagnosis despite the advancement of radiologic modalities because intra-operative findings may differ. Therefore, the confirmation of diagnosis was only made after surgery.

To our knowledge, there are only two previously published cases of concomitant unilateral maxillary FB and ACP. In this paper, we describe a third case ever reported about this finding. We reviewed the clinical, radiological, and pathological presentation of this rare entity, as well as the surgical management which was done.

The aim of this study is to spot the light of this rare presentation of simultaneous occurrence of two diseases together, ACP and FB, which may add to literature a theory for a fungal infection to be the causative agent of some types of ACPs.

## Conclusions

ACPs and FBs are common sinonasal diseases, but their concomitant occurrence is extremely rare. However, surgical management remains the mainstay of treatment, which can be achieved by multiple approaches, but the endoscopic sinus surgery is the best available modality.
